# Liver fluke in Irish sheep: prevalence and associations with management practices and co-infection with rumen fluke

**DOI:** 10.1186/s13071-019-3779-y

**Published:** 2019-11-06

**Authors:** Maria Pia Munita, Rosemary Rea, Ana Maria Martinez-Ibeas, Noel Byrne, Guy McGrath, Luis Enrique Munita-Corbalan, Mary Sekiya, Grace Mulcahy, Ríona G. Sayers

**Affiliations:** 1 Animal and Grassland Research and Innovation Centre (AGRIC), Teagasc, Moorepark, Fermoy, County Cork Ireland; 2 Department of Biological Sciences, Cork Institute of Technology, Bishopstown, Cork Ireland; 3 Centre for Veterinary Epidemiology and Risk Analysis, School of Veterinary Medicine, University College Dublin, Dublin, Ireland; 4 Generación Empresarial, Apoquindo, Las Condes, 6410 Santiago, Chile; 5 UCD, School of Veterinary Medicine, University College Dublin, Belfield, Dublin, Ireland

**Keywords:** *Fasciola hepatica*, Sheep, Prevalence, Co-infection, *Calicophoron daubneyi*, Breed, Treatment, Flukicide, Liver fluke

## Abstract

**Background:**

The present study aimed to identify the national prevalence of *Fasciola hepatica* in Irish sheep and to conduct a risk analysis assessment based on management and treatment practices in participating flocks. Also, co-infection with rumen fluke was quantified and its association with liver fluke and management practices was assessed.

**Methods:**

A total of 305 sheep flocks were selected ensuring even national representation of the sheep population. Participating farms were asked to complete a survey questionnaire on farm management practices and submit faecal samples during the winter of 2014–2015. Pooled faecal samples were analysed for the presence of *F. hepatica* and co-infection with rumen fluke. Apparent and true prevalence were calculated, additionally, the rate of co-infection with rumen fluke was also obtained. Correlation and regression analyses were used for assessing associations between management practices, liver fluke infection and co-infection with rumen fluke.

**Results:**

The national true prevalence of *F. hepatica* was 50.4% (*n* = 305). Regional prevalence varied from 41% in the east to 52% in the south. Co-infection with rumen fluke was observed in 40% of the studied population and correlated with increased *F. hepatica* egg counts (OR = 2.9; *P* ≤ 0.001). Predominant breeds were Suffolk, Texel and Horned Mountain breeds. Beef cattle were the most frequent type of other livestock present on farms and mixed species grazing was frequently reported (73%). More than half of the flocks reported a mid-to-late lambing period (March-April). Use of mountain land for grazing was of 32%. Flukicides were most commonly used twice over the autumn-winter period. Regression analyses highlighted significant association of *F. hepatica* status, with the presence of other livestock on farm, frequency of flukicides used during the winter and clinical presentation of liver fluke. A significant increase in eggs per gram of faeces was observed in Charollais sheep in comparison with all other breeds. Co-infection with *F. hepatica* and *Calicophoron daubneyi* was also significantly associated with the presence of other livestock on the farm, type of flukicide used and clinical fasciolosis.

**Conclusions:**

The present study provides up-to-date information on the prevalence of *F. hepatica* in Irish sheep and adds insight to the epidemiology of the disease. These findings will be useful for designing new holistic control measures for *F. hepatica* infection.

## Background

*Fasciola hepatica*, commonly known as the liver fluke, is a helminth parasite of mammals and a member of the Class Trematoda [1, 2]. It infects cattle, sheep, goat, horse, deer and humans as definitive hosts [3]. The parasite has a worldwide distribution and is considered an important disease of domestic livestock, especially in temperate climatic zones [4]. Fasciolosis has been estimated to account for annual losses of €90 million to the Irish livestock industry and €2.5 billion worldwide [5].

In sheep, liver fluke infection affects productivity and welfare [3]. It is a predisposing risk factor for mastitis [6] and drop in coagulation parameters [7]. The ingestion of large numbers of infective stages of the parasite can cause a highly pathogenic sub-acute presentation in lambs, characterised by hepatic haemorrhage and lesions, resulting in sudden death [1, 8]. Inflammatory mediators from liver damage might also affect early pregnancy [8]. Chronic fasciolosis, the most common clinical presentation, might lead to emaciation, especially in more susceptible animals and in ewes in the advanced stages of gestation [8]. In contrast to the dairy and beef sector, the cost of liver fluke in sheep enterprises is largely unquantified at a national and regional level [9].

*Fasciola hepatica* has an indirect life-cycle, with larval stages depending on a molluscan intermediate host for their development. The intermediate host species is largely determined by geographical location. In Europe, the most important snail in the fluke life-cycle is *Galba truncatula* [10]. In Ireland, *Radix* spp. and other genera have also been described as intermediate hosts, in addition to *G. truncatula* [11]. Temperature and moisture are the most important environmental factors for the presence of *G. truncatula* and *F. hepatica* development as wet soils with temperatures higher than 10 °C are required for their development [2]. The Irish climate provides ideal environmental conditions for *F. hepatica* in winter and early summer. The peak of infection in Irish sheep usually occurs in late winter and spring, following the summer infection of snails [8]. Conventionally control measures against *F. hepatica* chiefly rely on the use of anthelmintics. However, ideally, management practices and treatment should be used strategically, based on diagnosis- and evidence-based control measures for effectively reducing parasite burdens.

Paramphistomes, or rumen flukes, are represented by *Calicophoron daubneyi* and *Paramphistomum leydeni* in Ireland; however, the predominant species is *C. daubneyi* [12, 13]. Rumen flukes infect the same intermediate snail hosts as *F. hepatica* [14]. Infections of both rumen flukes and *F. hepatica* are acquired by ingestion of encysted metacercariae on grass. Probably the biggest difference between the two parasites is their pathology. Following ingestion and excystment, *F. hepatica* will migrate from the intestinal lumen, from the intestinal wall and peritoneum to reach the bile ducts for maturation [3], whereas immature paramphistome will attach to the small intestine mucosa for feeding before reaching the fore stomachs for maturation [15]. In contrast to *F. hepatica*, clinical paramphistomosis is rare, and is chiefly caused by large burdens of juveniles in the small intestine as adults in the forestomachs appear to be well tolerated [12, 15]. While *F. hepatica* can be treated with a range of flukicides, adult rumen flukes are only susceptible to oxyclozanide, with closantel being reported as having some efficacy against adult stages [16, 17]. This factor limits its control and increases the possibility of resistance as treatment rotations are not applicable [18]. The possibility that rumen fluke has adapted to the Irish climate more effectively than liver fluke, in addition to the fact that treatment against *F. hepatica* opens up the niche for paramphistomes, may result in the gradual replacement of *F. hepatica* by paramphistomes [19].

A previous *F. hepatica* pilot prevalence study in a small population of Irish sheep confirmed infection in 62% of animals [20], one of the highest recorded in Europe [9]. There are no up-to-date cross-sectional prevalence studies of *F. hepatica* in Irish sheep. Also, information on the relationship between *F. hepatica*, management factors and paramphistomes on a national scale is scarce. The present study aimed to generate national prevalence data for *F. hepatica* in Irish sheep flocks and to conduct a risk analysis based on management and treatment practices in participating flocks. Additionally, the study aimed to quantify the association and level of co-infection with rumen fluke.

## Methods

### Sample population

The present study was conducted between November 2014 and January 2015, coinciding with the high-risk period for fluke infection in the Irish temperate climate; 2014 was considered to be one of the warmest years in Ireland [21], probably benefiting the parasite. Flocks were recruited using Teagasc (Irish Food and Agriculture Development Authority) networks of Irish sheep farmers *via* 50 national Teagasc sheep advisors. Additionally, application forms were distributed through Teagasc Newsletters and the Irish Farmer’s Journal for circulation within farming and related communities. The application form consisted of a short questionnaire requesting the Teagasc advisor’s name, herd number, farmer’s name, postal address, GPS coordinates of the farmyard, mobile number, flock size (number of breeding animals), lambing season and preferred months of sampling. More than 350 applications were received. A total of 322 flocks were targeted, selected by stratified geographical location and flock size, to represent the national geographic spread according to the Census of Agriculture (2010) [22]. Once flocks were selected, farmers were informed by post and were requested to post back a consent form and dosing protocols. Consent forms were a prerequisite for taking part in the present study and allowed the use of farmer’s data in the study. The participation of farms was on a voluntary, non-incentivised basis.

### Sample collection

Sheep faecal samples were submitted between November 2014 and January 2015 to University College Dublin by post in a standardised kit [13], which briefly contained; 20 faecal containers (Sarstedt, Germany), a pre-paid postage envelope, an instruction leaflet and a sample submission form. Farmers were requested to obtain 20 fresh faecal catch samples from 20 different ewes in the flock and place each one in separate faecal container, this, together with flock sample size were determined using the Rogan-Gladen sample size estimator (http://www.ausvet.com.au). Samples were to be posted immediately after collection.

### Samples preparation and analyses

Upon receipt at the laboratory, faecal catch samples from each flock were pooled using 3 g of faeces from each pot, preparing two composite samples of 30 g, for representing all sampled animals. From each composite, 5 g of faeces were used to assess the number of liver fluke and rumen fluke eggs [3, 13]. Results from the faecal egg counts (FECs) were recorded as eggs per gram (epg) of faeces, assuming a test sensitivity of 90%.

### Questionnaire

A questionnaire was designed for the purpose of this study using a web-based survey tool (http://www.surveymonkey.com). This consisted of questions from a previous study [23] adapted for use on sheep farms. A total of 17 questions consisting of 12 multiple-choice, one ranking question and four open-ended questions, were organised in three sections: (i) farm background; (ii) *F. hepatica* management; and (iii) additional comments. The entire survey required approximately ten minutes to complete.

The form was reviewed by a group of sheep researchers based in Teagasc before being distributed by post to the participating farms. Prior to distribution, farmers received a text message informing them about the survey and a reminder text message was sent to farmers two weeks after distribution.

### Flock classification and management practices

The classification of *F. hepatica* status (positive or negative) was assigned based on the presence or absence of liver fluke eggs in pooled faecal samples. Classification of co-infection (observed or not observed) was assigned to flocks based on the presence or absence of both *F. hepatica* and rumen fluke eggs in the pooled samples.

Region (west, east and south) and flock size were obtained at recruitment or from the application form sent by the farmer. Classification by region was based on soil type according to Bloemhoff et al. [23]. Flock size was divided into two categories: < 120 or > 120 breeding animals (Table 1). Soil type self-classification was assigned into three categories: 1, dry; 2, damp; and 3, wet soil. Breeds included in the ‘other’ category (Table 1) included Belclare crosses, Lleyn crosses and mixed flocks with more than one predominant breed.

**Table 1:** Sheep flocks management variables and categories derived from survey, percentage of answers and negative and positive infection ratios

Question	Category	Answers % (*n*)	Negative % (*n*)	Positive % (*n*)
Breed	Horned mountain breed	11.9 (30)	5.9 (15)	5.9 (15)
	Suffolk and crosses	38.1 (96)	18.2 (46)	19.8 (50)
	Texal and crosses	24.4 (59)	11.1 (28)	12.3 (31)
	Cheviot	7.1 (18)	4.4 (11)	2.8 (7)
	Leicester and crosses	4.4 (11)	4.4 (11)	0 (0)
	Charollais and crosses	4.8 (12)	2.4 (6)	2.4 (6)
	Galway and crosses	0.4 (1)	0 (0)	0.4 (1)
	Other	9.9 (25)	5.9 (15)	3.9 (10)
Flock size	< 120	53.9 (138)	27.3 (70)	26.6 (68)
	> 120	46.1 (118)	26.6 (68)	19.5 (50)
Other livestock present on farm	None	25.0 (63)	15.1 (38)	9.9 (25)
	Beef	59.9 (151)	30.2 (76)	29.8 (75)
	Dairy	1.2 (3)	0.8 (2)	0.4 (1)
	Horses	3.6 (9)	0.8 (2)	2.9 (7)
	Other	10.3 (26)	5.2 (13)	5.2 (13)
Same paddock grazing of other livestock and sheep	No other livestock	24.5 (60)	13.9 (34)	10.6 (26)
	Same paddock grazing, not at same time	25.3 (62)	10.6 (26)	14.7 (36)
	Same paddock grazing, at same time	47.4 (116)	24.9 (61)	22.5 (55)
	No	2.9 (7)	1.6 (4)	1.2 (3)
Lambing period	December-January (early)	1.2 (3)	0.8 (2)	0.4 (1)
	January-March (early-mid)	14.9 (37)	6.9 (17)	8.1 (20)
	February-March (mid)	25.8 (64)	12.9 (32)	12.9 (32)
	March-April (mid-late)	52.4 (130)	27.4 (68)	25.0 (62)
	April-June (late)	3.2 (8)	2.8 (7)	0.4 (1)
	Other	2.4 (6)	1.2 (3)	1.2 (3)
Sheep grazing land	Only lowland	67.5 (168)	36.6 (91)	30.9 (77)
	Lowland (> 50%) and mountain	18.5 (46)	7.6 (19)	10.8 (27)
	Lowland and mountain (> 50%)	12.9 (32)	6.8 (17)	6.0 (15)
	Only mountain	1.2 (3)	0.8 (2)	0.4 (1)
Organic farm	Organic certified	2.9 (7)	1.2 (3)	1.6 (4)
	Organic not certified	7.3 (18)	3.3 (8)	4.1 (10)
	No	89.8 (221)	47.2 (116)	42.7 (105)

Details on management practices and dosing regimens were obtained *via* the questionnaire surveys. Classification of management practices (presence of other livestock in the farm, mixed species grazing, lambing period and mountain or lowland grazing, organic/conventional and slaughter plant feedback are shown in Tables 1 and 2.

**Table 2:** Liver fluke treatment variables and categories derived from survey, percentage of answers and negative and positive infection ratios

Question	Category	Answers % (*n*)	Negative % (*n*)	Positive % (*n*)
Illness or death due to liver fluke in last 5 years	Yes, multiple occasions	4.7 (12)	0.4 (1)	4.4 (11)
	Yes, rarely	34.0 (86)	15.4 (39)	18.6 (47)
	No	51.8 (131)	31.6 (80)	20.2 (51)
	Do not know	9.5 (24)	4.7 (12)	4.7 (12)
Liver fluke dosing regime	Do not dose	4.8 (12)	3.6 (9)	1.2 (3)
	Every month to six weeks in autumn	20.7 (52)	9.2 (23)	11.6 (29)
	Once over the autumn winter period	17.9 (45)	10.4 (26)	7.8 (19)
	Twice over the autumn winter period	49.4 (18)	25.1 (63)	24.3 (61)
	Other	7.2 (18)	4.0 (10)	3.2 (8)
Flukicides most commonly used	No flukicides used	3.2 (8)	2.4 (6)	0.8 (2)
	One adulticide	2.8 (7)	2.0 (5)	0.8 (2)
	Two adulticides	2.0 (5)	0.8 (2)	1.2 (3)
	One active against more than one stage	36.8 (91)	19.0 (47)	17.8 (44)
	More than one active against more than one stage	55.1 (136)	27.1 (67)	27.9 (69)
Flukicides used between September 2014 and April 2015	No flukicides used	0.9 (2)	0.4 (1)	0.4 (1)
	One maturicide	8.1 (18)	5.4 (12)	2.7 (6)
	Two maturicides	2.7 (6)	1.8 (4)	0.9 (2)
	One active against more than one stage	51.6 (115)	25.1 (56)	26.5 (59)
	More than one active against more than one stage	36.8 (82)	17.9 (40)	18.8 (42)
Flukicides frequency used between September 2014 and April 2015	0 times	0.4 (1)	0 (0)	1.0 (0.4)
	1 time	19.1 (44)	13.0 (30)	6.1 (14)
	2 times	39.8 (92)	21.7 (50)	18.2 (42)
	3 times	26.4 (61)	11.7 (27)	14.7 (34)
	4 times	13.4 (31)	4.8 (11)	8.7 (20)
	5 times	0.9 (2)	0 (0)	0.9 (2)
Administration of flukicides to animals separated in groups	No	93.0 (214)	48.3 (111)	44.8 (103)
	Yes	7.0 (16)	3.0 (7)	4.0 (9)
Product rotation	Same product every year	19.6 (48)	10.2 (25)	9.4 (23)
	Product rotation every year or every second year	62.0 (152)	29.8 (73)	32.2 (79)
	Use of any available product from veterinarian or licenced merchant	6.1 (15)	3.7 (9)	2.5 (6)
	Use of the cheapest or best deal product	3.7 (9)	2.0 (5)	1.6 (4)
	Use of product recommended by veterinarian	4.1 (10)	2.5 (6)	1.6 (4)
	No dosing	4.5 (11)	3.3 (8)	1.2 (3)
Information received from slaughter plant on liver fluke status	Yes, majority of animals with liver fluke evidence	1.7 (4)	0.4 (1)	1.3 (3)
	Yes, minority of animals with liver fluke evidence	25.8 (60)	12.0 (28)	13.7 (32)
	Never received liver fluke information from slaughter plants	72.5 (169)	38.6 (90)	33.9 (79)

### Treatment classification

Treatment management (Table 2) included: dosing frequency within the year of sampling, type of flukicides most commonly used, flukicides used in autumn and winter during the year of study and frequency of treatment during the period of sampling. Also, the use of treatment in different groups of animals and the rotation of flukicides were considered. Treatment variables derived from the questionnaire and categories are listed in Table 2.

Moreover, active ingredient of flukicides was considered as a variable and categorised as ‘used’ or ‘not used’ by each flock. This variable was created from ‘type of flukicides most commonly used’ answers.

### Statistical analyses

On receipt of the completed questionnaires, answers were manually entered into a web-based tool (http://www.surveymonkey.com), with the help and inspection of other researchers to verify correct data entry. Coded databases were downloaded into SPSS (IBM, USA) and used for initial descriptive analyses. Collation of the data and graphical representations were done with MS Excel (MS Office version 2010). A map was created in ArcGIS 10.3 © ESRI, Redland CA, using as backdrop the national sheep population in Ireland, based on the Department of Agriculture, Food and the Marine’s Ovine Census data, 2015. A kernel density estimation was applied, with a cell size of 100 metres and a search radius of 10 kilometres.

Apparent prevalence (Ap) was calculated based on the percentage of flocks recording positive FECs in the study. For the calculation of true prevalence (Tp) the Rogan-Gladen estimator in survey toolbox version 1.04 (http://www.ausvet.com.au) was used, assuming a test sensitivity of 90% and specificity of 99.9%. Prevalence was calculated both on a national and regional basis. Co-infection was calculated on the percentage of flocks where both parasites were detected in the composite sample.

Normality of the data was assessed visually using ladder of powers histograms, with normality of residuals assessed using normal probability plots and kernel density estimate plots constructed in Stata version 13 (StataCorp, USA). Pearson’s chi-square test was used for evaluating the univariable correlations between every categorical variable. Wilcoxon signed-rank test was applied for comparing the total sum of eggs per gram per region. Pearson’s chi-square, Wilcoxon test and final regression models were carried out using Stata 13. All regression models were constructed by completing a chi-squared univariable analysis examining all two-way associations. Those variables recording *P-*values of ≤ 0.15 in univariable analyses were included in multivariable models. A manual backwards elimination with a forward step was used to build models; some variables, based on the potential association with *F. hepatica*, were included in final models even if they did not show significance in the initial Pearson’s analysis (forced into the models). Both FEC categorisation (positive *vs* negative) and actual FEC were used as the categorical and continuous dependent variable for logistic and linear regression, respectively. Logistic regression was used for the co-infection model.

## Results

### Descriptive analyses

**Fig. 1:**
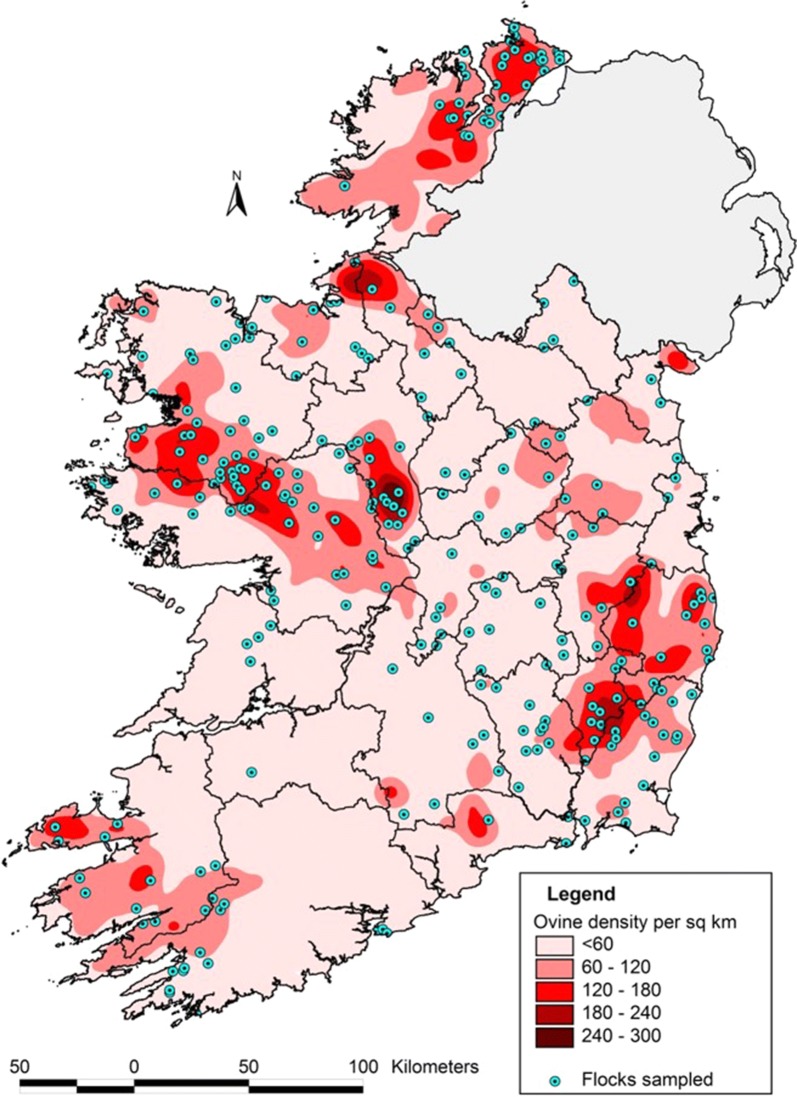
Map showing geographical distribution of participating flocks (blue dots) over national sheep density [22]

From the 322 flocks initially contacted a total of 305 flocks participated in the present study (Fig. 1). This yielded a sufficient sample size to achieve a 95% confidence level and precision of 5%, for a national sheep population of approximately 34,500 flocks with an expected national prevalence of 70%. The response rate for the survey was 83%, corresponding to 252 completed surveys.

The predominant breeds in the participating flocks were Suffolk and crosses (38.1%), Texal and crosses (24.4%) and Horned mountain (11.9%) (Table 1), accounting for 74.4% of flocks nationally. Each of the other specified breeds in the questionnaire (Cheviot and crosses, Leicester and crosses, Charollais and crosses and Galway and crosses) reported percentages below 10% (Table 1). Flocks classified as ‘other’ breeds showed the highest representation (56%) in the east region. Suffolk and Texal breeds showed the highest rates of *F. hepatica* infection (19.8% and 12.3%, respectively) (Table 1). Co-infection rates between breeds varied from 51% (Texal and crosses) to 0% (Leicester and Galway crosses) (data not shown).

Beef cattle were the most frequent type of ‘other livestock’ present on the same farm (Table 1), 25% of the farms did not report any other livestock. In general, the presence of other livestock on the same farm was not correlated with *F. hepatica* infection or co-infection with rumen fluke (*P* > 0.05) in the Chi-squared analysis (Additional file 1: Table S1).

Predominantly, grazing practices included a mix of species, 72.7% of the farms reported using the same grazing paddocks for different species, either at the same time (47.4%) or at different times (25.3%) (Table 1). Paddock grazing of sheep together with other livestock did not show any correlation with *F. hepatica* or co-infection (Additional file 1: Table S1).

More than half of the participating farms reported lambing between March and April (mid-late) (52%, *n* = 130) (Table 1), with lambing season correlated with geographical region (*P* = 0.012) (Additional file 1: Table S1), i.e. flocks located in the western part of the country were chiefly lambing during mid late season (35%).

Almost 70% of the flocks grazed on lowland only, while partial or complete use of mountain land for foraging was practiced by the remainder (Table 1). There was no correlation between this variable and infection with *F. hepatica* (Additional file 1: Table S1) or co-infection with rumen fluke. However, the grazing of mountain or low land pastures was correlated with region (*P* < 0.0001) and breed (*P* < 0.0001) (Additional file 1: Table S1). Flocks foraging in mixed low and mountain land were predominantly located in the west (23%) while flocks grazing only on mountain land were mostly located in the east and south (1.2%). Horned mountain and Cheviot breeds grazed primarily on mountain land and a small proportion of low land, with every other breed grazing mostly on low land pastures.

According to the farmers’ own classification of soil type in grazing areas, waterlogged zones were reported across all seasons. Most of the farms reported wetter land during winter and drier conditions in summer, with transitions during autumn and spring.

Only 10% of flocks were classified as organic (Table 1). 73% of the flocks reported never have received any liver fluke feedback from the slaughter house (Table 2).

### Liver fluke management and treatment practices

More than half of the participating flocks did not register critical illness or death due to liver fluke in the last five years (Table 2). This variable proved to be correlated with *F. hepatica* status (*P* = 0.002) and co- infection (*P* = 0.006) (Additional file 1: Table S1). Of the 131 flocks with no illness or death in the past five years, 80 were *F. hepatica*-negative according to faecal egg count.

The most common dosing regimen recorded was twice over the autumn and winter period (Table 2). Nine negative herds did not use any treatment (Table 2). The majority of *F. hepatica*-negative herds stated treating with flukicides once (10.4%) or twice (25%) over the autumn and winter period (Table 2). *Fasciola hepatica*-positive herds treated every month or six weeks during the autumn (12%) or twice during the autumn and winter period (24%) (Table 2). Nine *F. hepatica-* positive farms did not use any dosing treatment (Table 2).

**Fig. 2:**
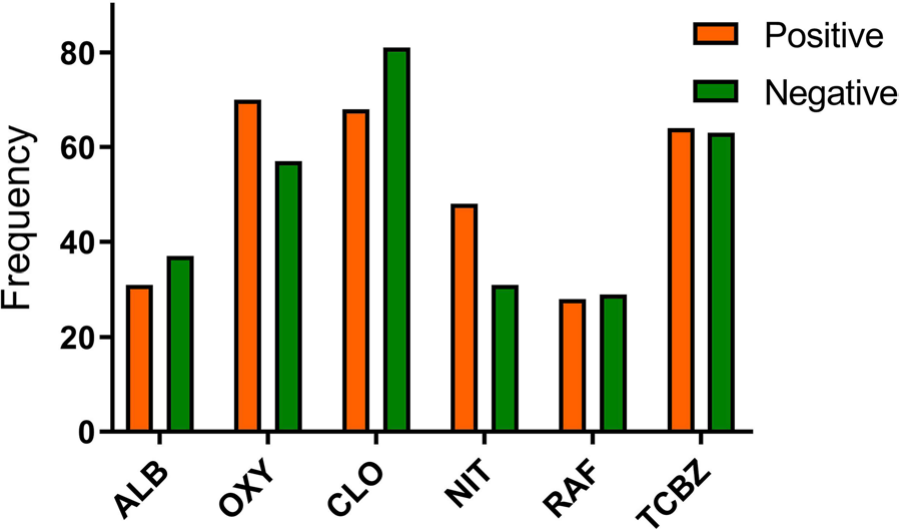
Bar graphic showing the frequencies of the flukicides most commonly used in positive and negative flocks. *Abbreviations*: ALB, albendazole; OXY, oxyclozanide; NIT, nitroxynil; RAF, rafoxanide; TCBZ, triclabendazole

The majority (92%) of farmers used flukicides active against immature as well as adult stages of the parasite (Table 2). This was true for both *F. hepatica*-negative and positive flocks. The most commonly used product was closantel, followed by oxyclozanide and triclabendazole. No significant differences between positive and negative flocks and the flukicides they used were observed. A bar graphic representing the frequencies of the most commonly used flukicides in positives and negative herds is shown in Fig. 2.

Flukicides used between March and April were most commonly products active against immature and mature flukes. The majority (85%) of these treatments were used between one and three times during this period (Table 2) with 46% in negative flocks and 39% in positive flocks, respectively. The type of flukicide used between March and April was correlated with treatment frequency (*P* ≤ 0.0001) and liver fluke status (*P* = 0.025) (Additional file 1: Table S1).

As shown in Table 2, 93% of the flocks did not treat animals in separated sub-groups. However, half of the flocks which treated in groups were located in the western region.

### Prevalence and co-infection

**Fig. 3:**
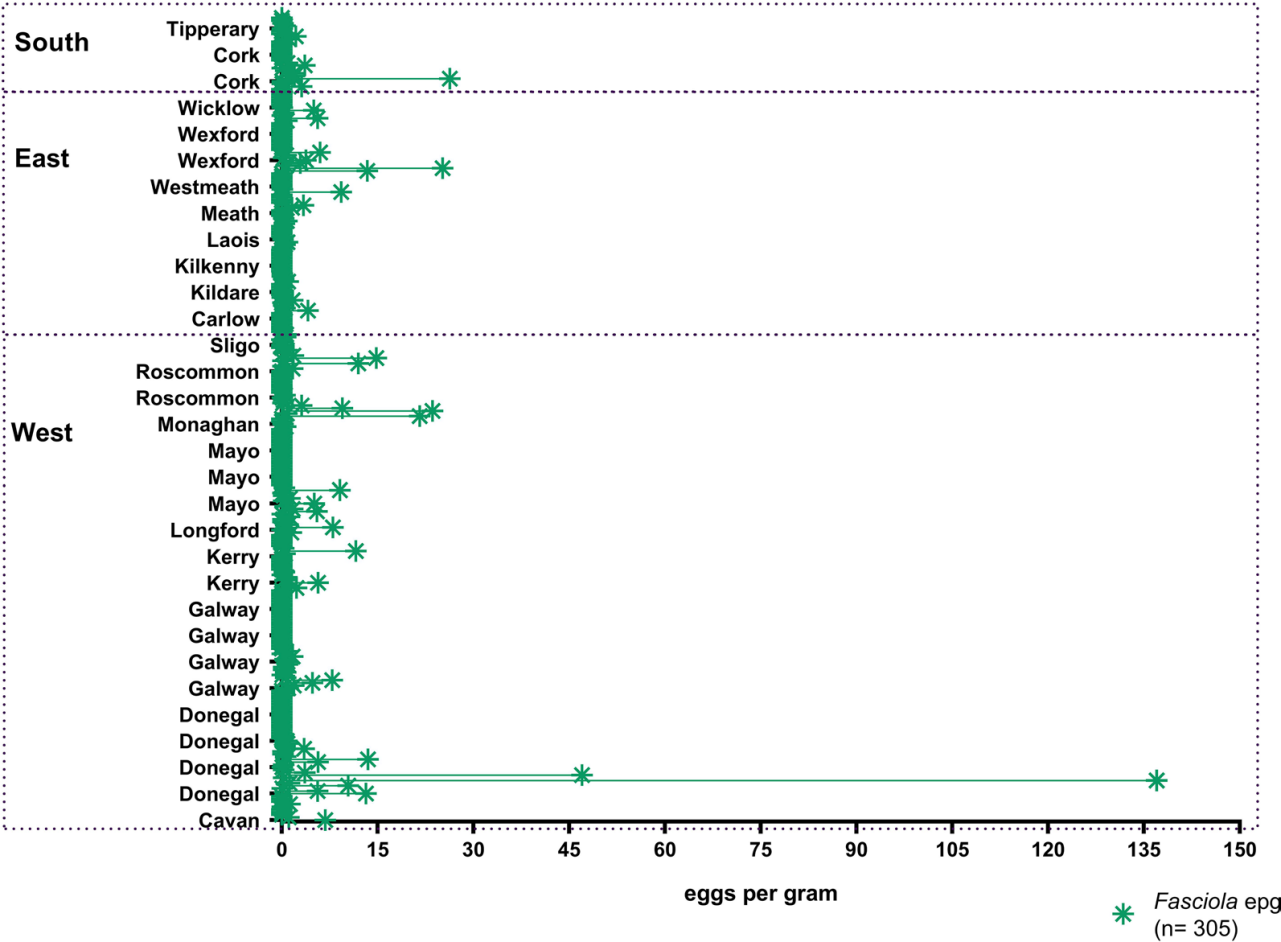
Dot plot representing *Fasciola hepatica* eggs per gram in counties and regions

*Fasciola hepatica* egg counts (Fig. 3) were not normally distributed and ranged between 0–137 epg. The highest egg counts and highest total sums of epg were recorded in the west of the country. The overall median was zero (0), as zero (0) was the most common faecal egg count registered, regional medians are shown in Table 3.

**Table 3:** Regional and national classification of eggs per gram (epg), total sum of epg, apparent prevalence (Ap), true prevalence (Tp), 95% confidence interval (CI), co-infection and flock size rate

	West (*n* = 183)	East (*n* = 96)	South (*n* = 26)	National (*n* = 305)
Median epg	0	0	0.1	0
Total sum of epg	428.6	95.4	39.7	563.7
Ap (%)	47.5	41.2	52.0	45.9
Tp (95% CI) (%)	53.1 (45.1–61.3)	45.7 (35.1–56.9)	55.1 (34.9–75.2)	50.4 (44.3–56.8)
Co-infection (%)	43.3	35.1	40.0	40.3
Flock size range	10–550	22–560	17–500	10–560

The national apparent prevalence (Ap) and estimated true prevalence (Tp) of *F. hepatica* were 45.9% and 50.4% (95% CI: 44.3–56.8%) (Table 3), respectively, assuming a test sensitivity of 90% and specificity of 99.9%. The prevalence across different regions varied from 41.2% in the east and 52% in the south (Table 3).

Paramphistome and *F. hepatica* co-infection was observed in 40.3% of the study population. Regionally, it ranged from 35.1% to 43.3% (Table 3). Only 17 flocks positive to *F. hepatica* did not show co-infection.

### Regression analyses

Logistic regression analysis identified the presence of horses (OR = 10.8, *P* = 0.035) in the participant farms as a risk factor for liver fluke infection, over flocks not sharing land with any other domestic animal species (Table 4). Infection status of horses present in participating farms were not recorded.

**Table 4:** Multivariable logistic regression of *F. hepatica* status (dependent variable) across other livestock present in farm, treatment count between September 2014 and April 2015 and illness or death due to *F. hepatica* (independent variables)

Independent variable	Odds ratio	95% CI	*P-*value	Model (*P-*value)
Other livestock present in farm				Other livestock present in farm *vs* treat count before sample *vs* illness or death due to liver fluke (*P* = 0.0020)
Horses *vs* none	10.78	1.18–98.37	0.035	
Horses *vs* beef	9.71	0.99–94.80	0.076*	
Treatment frequency before sampling				
1 treatment *vs* 2	0.48	0.21–1.08	0.077*	
1 treatment *vs* 3	0.32	0.13–0.78	0.012	
4 treatments *vs* 2	2.30	0.94–5.60	0.067*	
4 treatment *vs* 1	4.81	1.68–13.77	0.003	
Illness or death due to liver fluke				
Multiple occasions *vs* none	19.74	2.37–164.11	0.006	
Multiple occasions *vs* rarely	10.54	1.25–88.97	0.030	
Multiple occasions *vs* not known	12.92	1.33–125.86	0.028	

*Abbreviation*: CI, confidence interval

*Tendency

In general, treating with flukicides more than once in the period of sampling indicated a higher risk of *F. hepatica* in different degrees. As shown in Table 4, the use of one treatment prior to sampling decreased the risk of a positive FEC when compared to dosing two times (OR = 0.48, *P* = 0.077) or three times (OR = 0.32, *P* = 0.012). Also, dosing four times in this period, showed positive odd ratios against dosing twice (OR = 2.3, *P* = 0.067) or once (OR = 4.8, *P* = 0.003).

Presentation of multiple clinical cases in the past five years proved to be a predictive factor for liver fluke infection. The odds ratio of this variable against no clinical disease was almost 20 (*P* = 0.006) (Table 4). However, farms which reported no clinical episodes of *F. hepatica* presented higher risk (OR = 12.92, *P* = 0.028) than those reporting a clinical event rarely (OR = 10.54, *P* = 0.030) (Table 4).

Linear regression analyses of liver fluke egg counts showed that flocks co-infected with liver and rumen fluke presented higher *F. hepatica* egg counts (Coefficient = 2.9, *P* < 0.001) than flocks presenting liver fluke infection only (Table 5). Also, linear regression coefficients revealed higher eggs per gram values for Charollais flocks over flocks of any other breed. Suffolk and ‘other’ breed flocks showed higher eggs per gram counts than Horned mountain breed flocks (Table 5). Additionally, as shown in Table 5, flocks lambing between March and April (mid to late lambing season) showed an increase in almost 2 epg in comparison with flocks lambing between February and March (mid lambing season) (Coefficient = 1.97, *P* = 0.02). Also, the combined use of mountain and lowland for grazing increased the numbers of epg by 2.5 compared with flocks grazing lowlands only (Table 5).

**Table 5:** Multivariable linear regression of *F. hepatica* eggs per gram (dependent variable) across breed, treatment counts before sampling, flock size, lambing period, sheep grazing land, winter soil and illness or death due to *F. hepatica* (independent variables)

Independent variable	Coefficient	95% CI	*P-*value	Model (*P-*value)
Co-infection (observed *vs* not-observed)	2.90	1.44–4.35	< 0.001	
Breed				Co-infection *vs* breed *vs* treat count before sampling *vs* flock size *vs* lambing period *vs* grazing land type *vs* winter soil *vs* illness or death due to liver fluke (*P* = 0.0001)
Charollais *vs* mountain breed	7.70	3.48–11.93	< 0.001	
Charollais *vs* Suffolk	4.99	1.43–8.56	0.006	
Charollais *vs* Texal	5.13	1.47–8.79	0.006	
Charollais *vs* Cheviot	6.34	1.67–11.01	0.008	
Charollais *vs* Leicester	6.28	1.36–11.21	0.013	
Charollais *vs* Galway	12.79	1.93–23.64	0.021	
Charollais *vs* other breeds	4.79	0.71–8.87	0.022	
Mountain breed *vs* Suffolk	− 2.72	− 5.52–0.09	0.057*	
Mountain breed *vs* other breeds	− 3.17	− 6.63–0.29	0.073*	
Treatment frequency before sampling				
4 treatments *vs* 1	3.38	0.43–6.34	0.025	
4 treatments *vs* 2	3.65	1.18–6.12	0.004	
4 treatments *vs* 3	3.33	0.76–5.89	0.011	
Lambing period				
March-April (mid-late) *vs* February-March (mid)	1.97	0.31–3.65	0.021	
Sheep grazing land				
Lowland and mountain *vs* lowland	2.54	0.60–4.49	0.011	
Illness or death due to liver fluke in last 5 years				
No *vs* several No *vs* rarely	− 3.82 − 1.38	− 7.80–0.17 − 2.89– − 0.01	0.060* 0.072*	

*Abbreviation*: CI, confidence interval

*Tendency

As mentioned above, logistic regression highlighted increased risk of infection with the use of more than one treatment before sampling (Table 5). The same was observed in the continuous analysis, i.e. higher egg counts were positively correlated with treatment frequency (Table 5). Also, the manifestation of clinical disease showed a tendency for higher epg (Table 5), complementing the logistic regression results (Table 4).

Multivariable logistic regression for co-infection of liver and rumen fluke (Table 6) included flukicides most commonly used, summer soil type, other livestock present on farm and *F. hepatica* clinical presentation. There was an increased risk of co-infection (*P* = 0.051) in sheep flocks maintained with horses on the same farm, as was observed in the *F. hepatica* logistic regression (Table 4). Also, the presentation of illness or death due to liver fluke displayed higher odds ratios of co-infection than not presenting clinical infection. Finally, a tendency of higher risk of co- infection was observed in flocks commonly treated with flukicides in comparison with no treatment (Table 6).

**Table 6:** Multivariable logistic regression of liver fluke and rumen fluke co-infection (dependent variable) across flukicides most commonly used, summer grazing soil scale other livestock present in farm and illness or death due to *F. hepatica* (independent variables)

Independent variable	Odds ratio	95% CI	*P-*value	Model (*P-*value)
Flukicides most commonly used				Flukicides most commonly used *vs* summer soil type *vs* other livestock present in farm *vs* illness or death due to liver fluke (*P* = 0.0458)
More than one active against more than one stage *vs* no treatment	6.74	0.75–60.30	0.088*	
Other livestock present in farm				
Horses *vs* none	4.95	0.99–24.62	0.051*	
Illness or death due to liver fluke in last 5 years				
Multiple occasions *vs* rarely	5.49	1.09–27.53	0.039	
Multiple occasions *vs* no	8.54	1.75–41.70	0.008	
Multiple occasion *vs* not known	9.85	1.64–59.22	0.012	

*Abbreviation*: CI, confidence interval

*Tendency

## Discussion

There is no doubt of the impact that liver fluke can have on the health and welfare of ruminants, especially in temperate climatic zone like Ireland. The parasite also represents a major economic concern for ruminant production systems. Effective strategic control measures should be based on knowledge of local factors, incidence and management practices [24]. The present study aimed to determine the national prevalence of liver fluke in Irish sheep and to investigate its correlation with common farm management practices. In addition, inclusion of the national co-infection rate with paramphistomes facilitated investigation of risk factors for both trematodes. A previous study, conducted in a pilot area, reported a 62% liver fluke prevalence in Irish sheep [20]. This study represented just 7.1% of the national sheep population in the west of the country. The present study which included data collected in the whole country, estimated a national true prevalence of around 50%.

Recent liver fluke prevalence studies published elsewhere have reported infection rates of 41% in dairy cows in Switzerland [25], 64% in Mexico [26], and 57% in Poland [27]. These studies were based on bulk tank milk ELISA tests for the detection of *F. hepatica* exposure rather than active infection indicated by the presence of eggs in the faeces. The specificity of the FEC test is 100%, although, its sensitivity can be lower than 81% [28] and is dependent on the volume of sample analysed [29]. Therefore, the national prevalence in sheep flocks determined in the present study should be considered as a conservative estimate as the test only identifies sexually mature stages of the parasite.

The comparatively high prevalence reported in the present study can be explained by the temperate climate typical for Ireland as it provides optimum conditions for *G. truncatula* and the environmental stages of *F. hepatica* to thrive and infect ruminants. A higher total epg count was found in the West, with highest epgs seen in County Donegal (Fig. 3). In contrast the highest rate of infection was recorded in the south of the country (Table 3). The reason for this could be that the northern and western parts of the country are under a stronger maritime influence [30]. Other potential causes could be differences in treatments applied, as correlations were found between region, dosing regimens, frequency of treatment and other treatment variables (Additional file 1: Table S1). Although these effects were not detected in the regression analyses, it is possible that differences in regional treatments could have an effect in the number of eggs found. The high epg observed in County Donegal are alarming and indicate that the disease should be closely monitored in the region.

With regard to rumen fluke, a true prevalence of 86% was reported in the same study population [13] suggesting a relative competitive advantage of paramphistomes over *F. hepatica*, as they share the same intermediate host for completion of their life-cycle. A reason could be the frequent use of flukicides which are not effective against paramphistomes. It has been found that where *Fasciola gigantica* and paramphistomes co-occur, a larger proportion of animals excrete paramphistome eggs as compared to *Fasciola* eggs [31]. Yet, no differences in the prevalence of *C. daubneyi* and *F. hepatica* have been found in snail populations in France [32], However, in the UK, equivalent prevalence levels of *C. daubneyi* and *F. hepatica* within *G. truncatula* populations were associated with higher rumen fluke egg outputs and lower *F. hepatica* egg outputs from livestock grazing the snail habitats [33]. Rondelaud et al. [34] reported a faster development of one parasite over the other in co-infected *G. truncatula*, suggesting competition between these parasites in the intermediate host. Yet many questions remain to be answered regarding the relationship between paramphistomes, *F. hepatica* and their intermediate and final hosts. Further studies on the host competence of the various snails that occur in Ireland are also required and would add important information to the epidemiology of flukes under current and potential future environmental conditions.

A significant correlation was found between *Fasciola hepatica* and co-infection (*P* = 0.001), probably because the categorisation of one variable depended on the other and only 17 flocks were infected with *F. hepatica* alone. As both parasites share the same intermediate host, their development in the snail and infection of the final host is clearly linked. Additionally, the presence of co-infection increased *F. hepatica* FEC by 2.9 epg (P ≤ 0.001) and similar findings were observed in Welsh flocks [35].

As mentioned before, generally, most epidemiological studies on *F. hepatica* focus on dairy cows, yet, reports of risk factors associated with *F. hepatica* infection, intermediate and final hosts are currently limited. In the present study, the presence of other livestock on farm was a risk factor for infection with liver fluke (horses, *P* = 0.035) (Table 4) and co-infection with rumen fluke respectively (horses, *P* = 0.051) (Table 6). This has not been previously reported. Nonetheless, previous studies indicate the importance of determining ecological dynamics in multi-host parasite species [36–38]. Defining hosts-parasite interactions and identifying the hosts for the parasite [39, 40] would impact on control regimens applied to susceptible populations, as these factors contribute to the abundance and distribution of the disease. The susceptibility of horses to *F. hepatica* has been widely reported [3, 41, 42], and an abattoir study in Ireland, reported a *F. hepatica* prevalence of 9.5% in horses [43]. On the other hand, attempts to experimentally infect horses have failed [44, 45]. The findings of the present study and the literature strongly suggest the necessity for further investigations in the multi-host-parasite interactions for improvement in control measures. Additionally, these finding highlight the possible role of horses and other species, in the transmission of the liver fluke [13].

The majority of flocks enrolled in the present study were treated with flukicides with only 5% not using any type of flukicide for the control of *F. hepatica*. Although, differences between positive and negative flocks and flukicide were observed, these differences were not significant in the final correlation models. Most importantly, *F. hepatica* was present in the majority of the flocks regardless of treatment. Beesley et al. in 2017 [9] identified 20 reports of triclabendazole resistance in sheep within Europe. In Ireland, triclabendazole resistance has also been reported [46–48]. In contrast, other flukicides, such as nitroxynil [47] and closantel [49], seem to have retained their efficacy so far.

An important co-infection risk factor reported in the present study, was the use of triclabendazole. This result was expected, as this drug is not effective against paramphistomes. Closantel and oxyclozanide have shown to be effective parasiticides active only on adult paramphistome [16, 17]. Although this flukicide was commonly chosen by farmers (Fig. 2) in the present study, other flukicides such as; nitroxynil, rafoxanide and triclabendazole, were also considered within this variable, possibly justifying our findings.

Charollais and crosses sheep as a predominant breed in Irish flocks had increased eggs per gram per sample, in comparison to all other breeds. Remarkably, in Ireland this same effect was revealed in regard to rumen fluke [13]; however, Suffolk breed FECs showed to be significantly higher than other breeds. The susceptibility of Suffolk to helminth infections has been described in Ireland [50, 51] and internationally [52]. Nevertheless, no relationships between *F. hepatica* and Charollais breed have been found in the literature. However, increased genetic susceptibility in Charollais sheep to *Toxoplasma gondii* has been described [53] and also, increased sero-prevalence of *T. gondii* in Charollais lambs has been described [54]. Investigation on *F. hepatica* shedding of eggs in Charollais sheep should be carried out to confirm this finding.

As expected, the clinical presentation of *F. hepatica* correlated with infection (*P* = 0.002) demonstrating a good understanding of the clinical presentation by farmers and the proper diagnosis of the disease. This relationship was equally observed in multivariable *F. hepatica* and co-infection models.

At present, studies in the epidemiological aspects of *F. hepatica* in Irish sheep regarding the management and treatment practices are lacking. The present study estimated the prevalence of *F. hepatica* and its co-infection with rumen fluke based on recruiting a nationally representative flock population. Therefore, the results presented here are suitable for a better comprehension of the actual situation of these parasites in Ireland. The present study also provides a vision of the issues that require deeper knowledge for controlling fluke in sheep, especially under climate challenges.

## Conclusions

The present study provides a cross-sectional national insight into the prevalence of *F. hepatica* and co-infection with rumen fluke in sheep. Also, it provides risk factor analyses of management practices and dosing regimens. This study revealed high prevalence of the liver fluke in Irish sheep flocks. The co-infection of *F. hepatica* and rumen fluke was found to be associated with higher *F. hepatica* egg counts in sheep. Associations of liver fluke infection with horses present on farms and with Charollais breed are novel findings, although the implications of these outcomes remain to be elucidated. The increase of anthelmintic resistance worldwide has emphasized the importance of management strategies in parasite control and in that regard, the present study provides possible new lines of research in the presence of both trematodes for a holistic approach for the control of both diseases.

## Supplementary information

**Additional file 1.** Univariable Pearson’s chi-square analysis of independent categorical variables.

## Data Availability

All data are stored in the Teagasc (National Food and Development Authority) database. The datasets used and/or analysed during the present study are available from the corresponding author upon reasonable request.

## Abbreviations

epg: eggs per gram
Ap: apparent prevalence
Tp: true prevalence
