# Supplementary material for: Liver fluke in Irish sheep: prevalence and associations with management practices and co-infection with rumen fluke
Source: Parasit Vectors. 2019 Nov 6;12:525. doi: 10.1186/s13071-019-3779-y (PMC6836660; doi:10.1186/s13071-019-3779-y)
Supplement: Supplementary file 1 — Additional file 1. Univariable Pearson’s chi-square analysis of independent categorical variables. [file 13071_2019_3779_MOESM1_ESM.docx]

Additional file 1: Univariable Pearson’s chi-square analysis of independent categorical variables

|  | Fh | Fh-RF | Breed | Flock size | Region | Other livestock on farm | Same paddock graze | Lambing period | Sheep grazing land | Organic status | Spring soil | Summer soil | Autumn soil | Winter soil | Illness or death by Fh | Dosing regime | Most common flukicide | ALB | OXY | CLO | NIT | RAF | TCBZ | Dose before sampling | Treat count | Treat in groups | Flukicide rotation |
| --- | --- | --- | --- | --- | --- | --- | --- | --- | --- | --- | --- | --- | --- | --- | --- | --- | --- | --- | --- | --- | --- | --- | --- | --- | --- | --- | --- |
| Fh-RF | **0.001** |  |  |  |  |  |  |  |  |  |  |  |  |  |  |  |  |  |  |  |  |  |  |  |  |  |  |
| Breed | 0.057* | **0.038** |  |  |  |  |  |  |  |  |  |  |  |  |  |  |  |  |  |  |  |  |  |  |  |  |  |
| Flock size | 0.269 | 0.374 | 0.227 |  |  |  |  |  |  |  |  |  |  |  |  |  |  |  |  |  |  |  |  |  |  |  |  |
| Region | 0.491 | 0.420 | **0.021** | **0.023** |  |  |  |  |  |  |  |  |  |  |  |  |  |  |  |  |  |  |  |  |  |  |  |
| Other livestock on farm | 0.248 | 0.326 | 0.570 | **0.027** | 0.377 |  |  |  |  |  |  |  |  |  |  |  |  |  |  |  |  |  |  |  |  |  |  |
| Same paddock graze | 0.387 | 0.394 | 0.376 | **0.011** | 0.318 | **0.001** |  |  |  |  |  |  |  |  |  |  |  |  |  |  |  |  |  |  |  |  |  |
| Lambing period | 0.421 | 0.467 | **0.001** | **0.023** | **0.012** | 0.360 | 0.478 |  |  |  |  |  |  |  |  |  |  |  |  |  |  |  |  |  |  |  |  |
| Sheep grazing land | 0.441 | 0.559 | **0.001** | 0.186 | **0.001** | 0.105 | **0.015** | 0.137 |  |  |  |  |  |  |  |  |  |  |  |  |  |  |  |  |  |  |  |
| Organic status | 0.721 | 0.494 | **0.010** | 0.098 | 0.278 | 0.678 | 0.112 | 0.111 | 0.542 |  |  |  |  |  |  |  |  |  |  |  |  |  |  |  |  |  |  |
| Spring soil | 0.676 | 0.613 | 0.724 | 0.318 | 0.255 | 0.918 | 0.882 | 0.641 | 0.329 | 0.057* |  |  |  |  |  |  |  |  |  |  |  |  |  |  |  |  |  |
| Summer soil | 0.797 | 0.437 | 0.096 | 0.095 | 0.437 | 0.918 | 0.883 | 0.661 | **0.008** | **0.007** | **0.001** |  |  |  |  |  |  |  |  |  |  |  |  |  |  |  |  |
| Autumn soil | 0.521 | 0.890 | **0.041** | 0.110 | 0.754 | 0.064* | 0.485 | 0.204 | 0.850 | 0.203 | **0.001** | **0.001** |  |  |  |  |  |  |  |  |  |  |  |  |  |  |  |
| Winter soil | 0.816 | 0.758 | 0.187 | 0.091 | 0.350 | 0.897 | 0.856 | 0.144 | 0.815 | 0.552 | **0.001** | **0.001** | **0.001** |  |  |  |  |  |  |  |  |  |  |  |  |  |  |
| Illness or death by Fh | **0.002** | **0.006** | **0.021** | 0.498 | 0.072* | 0.439 | 0.589 | 0.710 | 0.625 | 0.782 | **0.001** | 0.760 | 0.887 | **0.045** |  |  |  |  |  |  |  |  |  |  |  |  |  |
| Dosing regime | 0.335 | 0.382 | **0.002** | 0.621 | **0.001** | 0.167 | **0.004** | 0.210 | **0.001** | **0.002** | 0.230 | 0.335 | 0.138 | **0.006** | 0.823 |  |  |  |  |  |  |  |  |  |  |  |  |
| Most common flukicide | 0.490 | 0.310 | 0.775 | **0.016** | 0.061* | 0.179 | 0.776 | **0.006** | **0.025** | 0.595 | 0.095 | 0.206 | 0.133 | **0.022** | 0.638 | **0.001** |  |  |  |  |  |  |  |  |  |  |  |
| ALB | 0.666 | 0.668 | 0.303 | 0.069* | 0.302 | 0.275 | 0.393 | **0.029** | 0.209 | 0.365 | 0.725 | 0.447 | 0.747 | 0.353 | 0.827 | 0.335 | **0.009** |  |  |  |  |  |  |  |  |  |  |
| OXY | **0.020** | **0.013** | 0.247 | 0.058* | 0.377 | 0.624 | 0.647 | 0.571 | **0.026** | 0.341 | 0.544 | 0.405 | 0.452 | 0.351 | 0.908 | **0.001** | **0.023** | **0.026** |  |  |  |  |  |  |  |  |  |
| CLO | 0.404 | 0.530 | **0.030** | **0.003** | **0.001** | **0.038** | **0.008** | 0.078* | 0.065* | 0.068* | **0.007** | 0.214 | 0.096 | **0.043** | 0.056* | **0.001** | **0.001** | 0.380 | 0.282 |  |  |  |  |  |  |  |  |
| NIT | **0.006** | 0.058* | 0.367 | 0.626 | 0.659 | **0.037** | 0.057* | 0.620 | 0.651 | 0.779 | **0.002** | 0.296 | 0.481 | 0.178 | **0.026** | 0.155 | **0.001** | 0.145 | 0.525 | 0.896 |  |  |  |  |  |  |  |
| RAF | 0.824 | 0.733 | **0.005** | 0.369 | 0.195 | 0.075* | 0.405 | 0.409 | **0.002** | 0.197 | 0.990 | 0.970 | 0.760 | 0.442 | **0.007** | **0.001** | **0.001** | 0.569 | 0.055* | 0.457 | 0.363 |  |  |  |  |  |  |
| TCBZ | 0.412 | 0.222 | 0.251 | **0.050** | 0.503 | 0.731 | 0.276 | 0.903 | 0.390 | 0.161 | 0.391 | 0.125 | 0.548 | 0.205 | 0.387 | **0.017** | **0.001** | **0.012** | 0.285 | 0.758 | 0.715 | 0.165 |  |  |  |  |  |
| Dose before sampling | 0.600 | 0.884 | 0.400 | **0.017** | **0.029** | **0.018** | 0.477 | 0.432 | **0.041** | 0.372 | **0.010** | **0.005** | **0.004** | **0.002** | 0.642 | **0.001** | **0.001** | **0.002** | 0.168 | **0.001** | **0.029** | **0.002** | **0.001** |  |  |  |  |
| Treat count | **0.025** | 0.102 | **0.003** | 0.197 | **0.045** | 0.910 | 0.542 | 0.246 | **0.001** | 0.751 | 0.061* | 0.165 | 0.157 | 0.081* | 0.242 | **0.001** | **0.001** | 0.200 | **0.001** | 0.066* | 0.154 | **0.032** | 0.171 | **0.001** |  |  |  |
| Treat in groups | 0.531 | 0.269 | 0.780 | 0.750 | 0.074* | 0.887 | 0.403 | 0.764 | **0.001** | 0.602 | 0.983 | 0.928 | 0.532 | 0.639 | 0.742 | **0.006** | 0.840 | 0.141 | 0.184 | 0.890 | 0.501 | 0.916 | 0.068* | 0.883 | 0.768 |  |  |
| Flukicide rotation | 0.622 | 0.277 | 0.195 | 0.433 | **0.013** | 0.971 | 0.717 | **0.014** | 0.777 | **0.001** | 0.898 | 0.223 | 0.921 | 0.595 | 0.700 | **0.001** | **0.001** | **0.001** | **0.001** | **0.007** | 0.128 | **0.021** | **0.001** | **0.001** | **0.001** | **0.013** |  |
| Factory comments | 0.391 | 0.424 | 0.480 | 0.211 | 0.812 | 0.835 | 0.933 | **0.001** | 0.931 | 0.122 | 0.350 | 0.445 | 0.273 | 0.124 | **0.001** | 0.078 | 0.585 | 0.750 | 0.617 | 0.122 | 0.317 | **0.040** | 0.696 | 0.450 | 0.531 | 0.834 | 0.468 |
